# The Conference Committees on Medical Unity

**Published:** 1902-12

**Authors:** 


A Monthly Review of Medicine and Surgery.

*EDITOR:*

WILLIAM WARREN POTTER, M. D.

All communications, whether of a literary or business nature, books for review and xchanges should be addressed to the editor: 284 Franklin Street, Buffalo, N. Y.

Vol. XLII.—LVIII. DECEMBER, 1902. No. 5.

### The Conference Committees on Medical Unity.

THE report of the deliberations of the committees of conference of the state society and state association as published in the *New York State Journal of Medicine* for November contains important information which appears to justify comment at this time. It will be remembered by those interested that the committees were appointed for the purpose of "the reorganisation of the regular medical profession of this state," as suggested by Dr. Henry L. Elsner in his inaugural address at the 96th annual meeting of the state society. The net result of the conferences, of which there have been several, seems to be that the society and the association, chartered in 1806 and in 1900 respectively, are expected to unite in a petition to the next legislature asking that their existing charters be repealed and that they be reorganised into a society to be known as The Medical Society of the State of New York. Section 1 of the proposed act provides, "There shall be established by the physicians named in section six of this act an organisation styled The Medical Society of the State of New York."

The reason why the state society should give up its charter, rights, and existence, is set forth in the following: the said society was "formed under a law of 1806, was changed materially in 1813, after which nearly all the important privileges granted have been repealed by many subsequent acts of the legislature, so that the basis of its existence is, today, so involved as to be little understood." This misleading statement which seems to come from the committee of the association, is made and repeated, does not appear to have been questioned by anyone, and stands as the alleged excuse for the abolition of the state society in its 98th year. Of this statement it may be observed that its element of truth is in the assertion as to when the state society was formed—namely, in 1806. Its further allegations, all and singular, are also misleading in effect. To call attention to the fact and to make the assertion good, is the purpose of this writing. Fortunately, the issue in this case is not decided by shifting emphasis, forcing construction, or straining interpretation. The question is most simple. Do or do not the statutes of 1806 and 1813 exist? Are the privileges of the county societies and state society of "today" less, or equal, or greater than those of former times?

Again, events have, fortunately, conspired to make the proof in this issue not only easy but conclusive. The laws of the state have recently been quite thoroughly revised by a committee on statutory revision. This body of eminent men concluded its labors in 1895, and as a result of its advice and recommendations the entire medical law of the state has been revised and re-enacted. As re-enacted, and as the law of "today" the essential sections of 1806 and 1813, giving powers and privileges to state and county societies, stand in the identical words of the original acts.[1]

The only privileges which were repealed related to the examination of resident students, who were unable to attend a medical college, and who desired to practise medicine within the state. The authority to examine and license these students was without doubt helpful in establishing the popularity and influence of the county societies; but the increase in the number of the medical colleges soon made the practice obsolete and at the time of the repeal of the sections in 1887, they had long been inoperative. However, this loss of power on the part of the organised profession was but a shift of responsibility from one medical body to another, from the society to the college. It was but temporary, for in 1890 the state examining board was authorised as an agent of the state society, and to that board was given powers a hundred fold more important than those repealed in 1887, or at any or all previous times. From this time, 1890, the organised medical profession has guarded the roads leading to the practice of that profession in the state and examined each and every applicant considered fit for examination.

1. Revised Statutes, Codes, and General Laws of the State of New York. Second edition. By Clarence F. Birdseye. Vol. II. 1896. Page, 1968.

Again, in 1885, the state society was given authority to regulate its own membership, whereby it entered upon a condition of independence never before enjoyed, thus gaining the ability to receive delegates, as it now does, from voluntary medical societies, and also ability to increase its number of delegates from the county societies to five times the usual number. To these enormous and newer powers it should also be added that the county society still has, as it has had since 1819, authority to collect annual dues from every physician residing within the county.

All the foregoing conditions are plainly and unquestionably provided for in the various sections of the medical law of "today" and many, even most of them, have been in continuous existence since 1806. We characterised the statement of the committee of the association as misleading which in view of the foregoing statement of facts seems just.

These, then, are some of the reasons why we deem it inexpedient at this time to abolish the charter, the birthright, of our state society which for nearly a century has been the exponent of the organised medical profession of the state.

But if we must express our decided opposition to abolishing our charter, we are not opposed to its amendment from time to time to meet changed and ever changing conditions as they arise or may arise. "Thus far shalt thou go and no farther."

